# Access to Mental Health and Substance Use Treatment in Comprehensive Primary Care Plus

**DOI:** 10.1001/jamanetworkopen.2024.8519

**Published:** 2024-04-26

**Authors:** Tatiane Santos, Alon Bergman, Aaron Smith-McLallen

**Affiliations:** 1 Tulane University School of Public Health and Tropical Medicine, New Orleans, Louisiana; 2 Leonard Davis Institute of Health Economics, University of Pennsylvania, Philadelphia; 3 Department of Medical Ethics and Health Policy, Perelman School of Medicine, University of Pennsylvania, Philadelphia; 4 Health Care Management Department, The Wharton School, University of Pennsylvania, Philadelphia; 5 Independence Blue Cross, Philadelphia, Pennsylvania

## Abstract

**Importance:**

To meet increasing demand for mental health and substance use services, the Centers for Medicare & Medicaid Services launched the 5-year Comprehensive Primary Care Plus (CPC+) demonstration in 2017, requiring primary care practices to integrate behavioral health services.

**Objective:**

To examine the association of CPC+ with access to mental health and substance use treatment before and during the COVID-19 pandemic.

**Design, Setting, and Participants:**

Using difference-in-differences analyses, this retrospective cohort study compared adults attributed to CPC+ and non-CPC+ practices, from January 1, 2018, to June 30, 2022. The study included adults aged 19 to 64 years who had depression, anxiety, or opioid use disorder (OUD) and were enrolled with a private health insurer in Pennsylvania. Data were analyzed from January to June 2023.

**Exposure:**

Receipt of care at a practice participating in CPC+.

**Main Outcomes and Measures:**

Total cost of care and the number of primary care visits for evaluation and management, community mental health center visits, psychiatric hospitalizations, substance use treatment visits (residential and nonresidential), and prescriptions filled for antidepressants, anxiolytics, buprenorphine, naltrexone, or methadone.

**Results:**

The 188 770 individuals in the sample included 102 733 adults (mean [SD] age, 49.5 [5.6] years; 57 531 women [56.4%]) attributed to 152 CPC+ practices and 86 037 adults (mean [SD] age, 51.6 [6.6] years; 47 321 women [54.9%]) attributed to 317 non-CPC+ practices. Among patients diagnosed with OUD, compared with patients attributed to non-CPC+ practices, attribution to a CPC+ practice was associated with filling more prescriptions for buprenorphine (0.117 [95% CI, 0.037 to 0.196] prescriptions per patient per quarter) and anxiolytics (0.162 [95% CI, 0.005 to 0.319] prescriptions per patient per quarter). Among patients diagnosed with depression or anxiety, attribution to a CPC+ practice was associated with more prescriptions for buprenorphine (0.024 [95% CI, 0.006 to 0.041] prescriptions per patient per quarter).

**Conclusions and Relevance:**

Findings of this cohort study suggest that individuals with an OUD who received care at a CPC+ practice filled more buprenorphine and anxiolytics prescriptions compared with patients who received care at a non-CPC+ practice. As the Centers for Medicare & Medicaid Innovation invests in advanced primary care demonstrations, it is critical to understand whether these models are associated with indicators of high-quality primary care.

## Key Points

**Question:**

Was the Comprehensive Primary Care Plus (CPC+) demonstration associated with improved access to mental health and substance use services?

**Findings:**

In this cohort study of 188 770 adults with anxiety, depression, or opioid use disorder (OUD), findings were mixed. Individuals with OUD who received care at CPC+ practices filled more buprenorphine and anxiolytic prescriptions than those receiving care at non-CPC+ practices.

**Meaning:**

Findings of this study suggest that integration of behavioral health in primary care may improve access to mental health and substance use treatment, especially for individuals with OUD.

## Teaser

This cohort study examines whether participation in the Centers for Medicare & Medicaid Services’ Comprehensive Primary Care Plus model was associated with improved access to mental health and substance use treatment.

## Introduction

The COVID-19 pandemic has deeply impacted population mental health, which has worsened since the start of the pandemic.^1^ Among adults aged 18 years and older, the prevalence of depressive symptoms was 27.8% in 2020 and increased to 32.8% in 2021. Overall, the share of all outpatient visits associated with a mental health or substance use diagnosis grew from 4% in 2019 to 8% in 2020 and 2021.^2^ Complicating matters, the US has experienced a shortage of mental health care professionals that predates the pandemic, and the shortage is projected to worsen.^3^ Recent data show that approximately 27% of adults who reported symptoms of anxiety or depression during the pandemic also reported an unmet need for counseling or therapy.^4^

Behavioral health integration into primary care is a promising way to meet the increasing demand for mental health and substance use services. There are several approaches to behavioral health integration into primary care, with most sharing common elements, including screening for depression, anxiety, and other behavioral disorders using validated instruments; team-based care with nonphysician staff; and shared information systems to facilitate coordination across health care professionals. Another common feature of these approaches is their use of behavioral health providers, including behavioral health consultants, nurse practitioners, physician assistants, substance use and mental health counselors, and social workers. By leveraging other behavioral health providers, these models seek to amplify access to mental health and substance use services and to improve patient outcomes while decreasing the cost of care.

Behavioral health integration into primary care can effectively improve depression and anxiety symptoms, physical and mental health quality of life, social role functioning, and patient satisfaction compared with usual care.^5,6^ A promising model of behavioral health integration into primary care was the Comprehensive Primary Care Plus (CPC+), an advanced primary care medical home model implemented from 2017 to 2021 and sponsored by the Centers for Medicare & Medicaid Innovation (CMMI).^7^ A recent study found that CPC+ practice participation was associated with reduced outpatient emergency department visits and acute hospitalizations.^8^ These reductions were not associated with changes in Medicare expenditures, as decreases in acute inpatient care spending were offset by spending increases on other services, such as inpatient rehabilitation facilities and physician and nonphysician noninstitutional services.^8,9^

As the demand for mental health and substance use services continues to increase, it is important to understand whether behavioral health integration into primary care can alleviate barriers to care and improve patient outcomes. This study examined the association of participation in the CPC+ demonstration with total cost and access to mental health and substance use treatment. To our knowledge, this is the first study to examine the association of CPC+ participation with access to mental health and substance use treatment in the periods before and after COVID-19 was declared a public health emergency (PHE).

## Methods

This cohort study was approved by the Tulane University School of Public Health’s institutional review board. The need for informed consent was waived because we used deidentified secondary data. This study followed the Strengthening the Reporting of Observational Studies in Epidemiology (STROBE) reporting guideline.^10^

### Data

#### Comprehensive Primary Care Plus Demonstration

The goal of the CPC+ demonstration was to improve quality, access, and efficiency of primary care through multipayer payment reform and care delivery transformation.^7^ Practices participating in CPC+ implemented several care delivery changes, including access and continuity measures, care management, comprehensive and coordinated care, patient and caregiver engagement, and data-driven population health management. There were 2 tracks in CPC+, and practices in track 2 were required to implement more advanced transformation measures. While all CPC+ practices were required to integrate behavioral health services by 2019, practices in track 2 did so 1 year before practices in track 1. Practices selected from 2 approaches or a combination thereof to integrate behavioral health: the primary care behaviorist model or the care management for mental illness model, with the majority of participating practices implementing the former.^11^ To support practice transformation, CMMI provided per-member-per-month risk-adjusted care management fees and performance-based incentives. Practices in track 2 also received prospective quarterly payments along with lower Medicare fee-for-service reimbursement. A detailed description of CPC+ requirements is available elsewhere.^11^

#### Member Attribution

This study analyzed data from Independence Blue Cross (Independence), a large commercial insurer in Philadelphia, Pennsylvania. The sample was limited to Independence’s commercial insurance plans. Independence provided a list of practices that participated in CPC+. A total of 178 practices participated in CPC+ and 5430 practices did not participate. We excluded practices that were not part of the network throughout the study period from 2018 to 2022. The final list of clinicians was merged with the member eligibility file using unique provider identifiers to identify members attributed to CPC+ clinicians and non-CPC+ clinicians. Members who had a health maintenance organization plan were attributed to the primary care physician (PCP) they selected. For all other members, we used Independence’s PCP attribution methodology, which requires at least 2 PCP visits over the past 12 months. In the case of a tie in the number of visits to different PCPs, members were attributed to the PCP for their most recent visit. This study used an intention-to-treat approach; that is, we used the earliest member PCP attribution, and patients remained with the same PCP throughout the study period.

#### Patient-Level Inclusion and Exclusion Criteria

We used deidentified data from medical and pharmacy claims and patient eligibility files from January 1, 2018, to June 30, 2022. We restricted the sample to patients aged 19 to 64 years who resided in Independence’s main geographical areas covering 5 contiguous Pennsylvania counties (Bucks, Chester, Delaware, Montgomery, and Philadelphia), had 12 months of continuous enrollment, and had any diagnosis of depression, anxiety, or opioid use disorder (OUD) in the baseline period (2018-2019). After applying these exclusion criteria, the study sample included 188 770 patients, with 102 733 patients attributed to 152 CPC+ practices and 86 037 attributed to 317 non-CPC+ practices.

### Primary Outcomes and Covariates

Primary outcomes were defined at the per-patient-per-quarter level and included total cost of care and the number of PCP visits for evaluation and management, community mental health center visits, psychiatric hospitalizations, substance use treatment visits (residential and nonresidential), and prescriptions filled for antidepressants, anxiolytics, buprenorphine, naltrexone, or methadone. We used place of service from the claims data to identify the site of care. We used the Healthcare Common Procedure Coding System and *Current Procedural Terminology* codes to identify PCP evaluation and management visits (eTable 1 in Supplement 1). Antidepressant, anxiolytic, buprenorphine, naltrexone, and methadone prescriptions were identified using National Drug Codes (NDCs) from the US Food & Drug Administration NDC Directory.^12^ To account for changes to NDC codes over time, we used brand name and generic names to capture NDCs that were not included in the most current NDC Directory (eTables 2-6 in Supplement 1). Total cost was calculated by adding all paid claims in a given quarter per patient excluding out-of-pocket expenses. Study covariates included patient age and comorbidities (heart failure, ischemic heart disease, hypertension, hyperlipidemia, chronic obstructive pulmonary disease, diabetes, chronic kidney disease, and rheumatoid arthritis or osteoarthritis).

### Statistical Analysis

We used a difference-in-differences multivariate model to estimate the association between CPC+ participation and patient outcomes using generalized least-squares regression. Study periods were defined based on the date that COVID-19 was declared a PHE (March 14, 2020). To account for the disruptions to health care utilization around the time that COVID-19 was declared a PHE, we excluded observations that covered 10 weeks before and 10 weeks after declaration of the PHE.^13^ These effects were estimated using the following model:

*Y*_ijt_ = α + θ*CPC*_j_ × *Post*_t_ + β*member*_ijt_ + γ_i_ + *Q*_t_ + ε_ijt_

The outcome variable, *Y*_ijt_, is defined for member *i* attributed to PCP *j* during quarter *t*. The key explanatory variable is θ, which is the difference-in-differences estimator and measures the association between CPC+ practices and patient outcomes vs non-CPC+ practices (ie, risk difference). In this equation, β is a vector for patient-level covariates. Additionally, the model controls for patient (*Y*_i_) and quarter (*Q*_t_) fixed effects. Quarter fixed effects help account for secular trends, while patient fixed effects control for unobserved, time-invariant patient-level characteristics. The error term ε_ijt_ represents the remaining, unobserved variation in patient and practice characteristics. Standard errors were clustered at the provider level.

We estimated separate models for 2 groups of patients: (1) those diagnosed with depression or anxiety and (2) those diagnosed with OUD. Finally, to understand whether patient outcomes changed in the context of continued pandemic challenges, we estimated the impact of CPC+ in each posttreatment year separately.

We tested for pretreatment parallel trends for all primary study outcomes by limiting the sample to the pretreatment period (2018-2019), and tested the significance of the interaction between the linear quarter dummies and the treatment variable. Statistical significance was set at 2-sided *P* < .05 and a nonsignificant *P* value indicates that the pretreatment trends are parallel. All analyses were conducted from January to June 2023 using Stata, version 17 (StataCorp LLC).

To enroll in CPC+, practices had to meet certain requirements, and some practices that met the requirements chose not to participate in CPC+.^7,14^ As a result of both enrollment requirements and self-selection into the program, CPC+ practices could look inherently different from other practices.^14^ A valid comparison of CPC+ practices with non-CPC+ practices must account for baseline differences in practice characteristics. In this analysis, we used an inverse probability weighting (IPW) method to balance the sample. We first performed a probit regression to estimate the probability of a practice enrolling in CPC+ as a function of practice size (physicians and patients), percentage of male patients, patient age, and insurance type. We then weighted observations in the estimation of the equation above using the IPW calculated using propensity scores. We omitted the bottom and top 5% of the sample based on the predicted inverse probability weights. The eMethods in Supplement 1 provides more details on the IPW approach used.

## Results

The final sample included 469 practices and 188 770 members. A total of 102 733 patients (mean [SD] age, 49.5 [5.6] years; 57 531 women [56.4%] and 45 202 men [43.6%]) were attributed to 152 CPC+ practices, and 86 037 patients (mean [SD] age, 51.6 [6.6] years; 47 321 women [54.9%] and 38 716 men [45.1%]) were attributed to 317 non-CPC+ practices (Table 1). Overall, CPC+ practices were larger in terms of the number of affiliated physicians and attributed members. These practices also had a lower proportion of patients diagnosed with physical chronic conditions but a similar distribution of patients diagnosed with behavioral health conditions compared with the non-CPC+ practices. Patients in CPC+ practices were more likely to have preferred provider organization coverage and less likely to have health maintenance organization coverage. Before IPW, there was a statistically significant difference in means between the 2 groups across most practice characteristics (Table 2). After IPW, the balance of characteristics across the 2 groups was substantially improved (Table 2).

**Table 1.** Baseline Practice Characteristics for CPC+ Practices and Non-CPC+ Practices^a^

Characteristic	Non-CPC+ practices (n = 317)	CPC+ practices (n = 152)	*P* value
Practice size			
No. of physicians, mean (SD)	4.4 (5.5)	7.5 (8.5)	<.001
No. of members, mean (SD)	1135.7 (964.1)	2181.6 (1509.5)	<.001
Patient demographics			
Female, No. (%)	47 321 (54.9)	57 531 (56.4)	.06
Male, No. (%)	38 716 (45.1)	45 202 (43.6)	.06
Age, mean (SD), y	51.6 (6.6)	49.5 (5.6)	.001
Patients aged ≥65 y, No. (%)	23.7 (14.2)	19.6 (12.2)	.003
Patient physical chronic conditions, mean (SD), %			
Hyperlipidemia	46.7 (15.2)	37.7 (11.2)	<.001
Hypertension	42.8 (15.1)	32.9 (11.5)	<.001
Rheumatoid arthritis or osteoarthritis	26.3 (10.6)	21.1 (7.0)	<.001
Diabetes	18.9 (13.8)	11.2 (5.3)	<.001
COPD	9.1 (5.2)	7.8 (4.4)	.006
Ischemic heart disease	10.1 (6.7)	7.1 (4.3)	<.001
Chronic kidney disease	8.7 (7.6)	5.8 (4.6)	<.001
Heart failure	5.6 (6.3)	3.7 (4.1)	<.001
Patient behavioral chronic conditions, mean (SD), %			
Anxiety disorders	19.3 (6.8)	21.2 (3.9)	.001
Depressive disorders	13.3 (5.4)	14.2 (3.6)	.08
Drug use disorders	4.0 (2.6)	3.3 (1.4)	.003
Opioid use disorders	1.7 (1.5)	1.3 (0.8)	.003
Alcohol use disorders	2.5 (1.3)	2.2 (0.6)	.010
Bipolar disorders	2.2 (2.3)	2.2 (0.9)	.98
Schizophrenia and other psychotic disorders	1.0 (1.1)	0.8 (0.9)	.08
Patient insurance coverage, mean (SD), %			
PPO	44.5 (12.7)	50.1 (11.9)	<.001
HMO	57.7 (13.1)	53.9 (10.6)	.002
Health care utilization by site of care, mean (SD), No. of visits/1000 patients/quarter			
Total spending, $/1000 patients/quarter	10 206 000 (32 974 000)	10 006 000 (34 678 000)	<.001
PCP evaluation and management visits	382 (1177)	324 (977)	<.001
Psychiatric hospitalizations	22 (1012)	19 (977)	<.001
Community mental health center visits	7 (341)	1 (148)	<.001
Substance use treatment (residential and nonresidential combined), mean (SD), No. of prescriptions	27 (1374)	10 (513)	<.001
No. of prescriptions/1000 patients/quarter, mean (SD)			
Antidepressants	807 (1525)	1005 (1659)	<.001
Anxiolytics	437 (1148)	433 (1138)	<.001
Buprenorphine	43 (426)	28 (356)	<.001
Methadone	4 (112)	6 (138)	<.001
Naltrexone	5 (126)	5 (120)	.15

Abbreviations: COPD, chronic obstructive pulmonary disease; CPC+, Comprehensive Primary Care Plus; HMO, health maintenance organization; PCP, primary care practitioner; PPO, preferred provider organization.

^a^ Baseline characteristics were calculated using baseline data from 2019. The data presented are for the final unweighted study sample.

**Table 2.** Covariate Balance After Inverse Probability Weighting^a^

Covariate	Sample before IPW (n = 469 practices)		Sample after IPW (n = 469 practices)	
	Standardized difference (SE)	*P* value	Standardized difference (SE)	*P* value
Practice size				
No. of physicians	0.46 (0.11)	<.001	0.03 (0.10)	.72
No. of members	0.83 (0.11)	<.001	−0.17 (0.16)	.27
Patient demographics				
Female	0.19 (0.10)	.05	0.02 (0.13)	.85
Male	−0.19 (0.10)	.05	−0.02 (0.13)	.85
Age	−0.32 (0.10)	<.001	0.00 (0.16)	>.99
Age ≥65 y	−0.30 (0.10)	.001	0.01 (0.16)	.96
Patient physical chronic conditions				
Hyperlipidemia	−0.62 (0.08)	<.001	−0.22 (0.14)	.13
Hypertension	−0.67 (0.08)	<.001	−0.32 (0.13)	.02
Rheumatoid arthritis or osteoarthritis	−0.52 (0.08)	<.001	−0.13 (0.20)	.51
Diabetes	−0.62 (0.07)	<.001	−0.33 (0.11)	.002
COPD	−0.27 (0.09)	.004	−0.02 (0.13)	.88
Ischemic heart disease	−0.48 (0.08)	<.001	−0.18 (0.12)	.14
Chronic kidney disease	−0.42 (0.08)	<.001	−0.06 (0.16)	.72
Heart failure	−0.34 (0.08)	<.001	−0.01 (0.18)	.97
Patient behavioral chronic conditions				
Anxiety disorders	0.32 (0.08)	<.001	0.24 (0.09)	.007
Depressive disorders	0.18 (0.09)	.04	0.12 (0.10)	.25
Drug use disorders	−0.30 (0.08)	<.001	−0.21 (0.11)	.06
Opioid use disorders	−0.29 (0.08)	<.001	−0.17 (0.10)	.08
Alcohol use disorders	−0.25 (0.08)	.001	−0.12 (0.11)	.28
Bipolar disorders	0.00 (0.08)	.97	0.02 (0.07)	.79
Schizophrenia and other psychotic disorders	−0.17 (0.09)	.06	−0.07 (0.08)	.42
Patient insurance coverage				
PPO	0.44 (0.09)	<.001	0.23 (0.15)	.11
HMO	−0.31 (0.09)	<.001	−0.15 (0.14)	.30

Abbreviations: COPD, chronic obstructive pulmonary disease; HMO, health maintenance organization; IPW, inverse probability weighting; PPO, preferred provider organization.

^a^ Differences were calculated from standardized variables using baseline data from 2019, comparing the final study sample before and after IPW. To get the final study sample, the raw sample (ie, all primary care practices) was trimmed by omitting the bottom and top 5% of the sample in terms of IPW. The raw sample had a total of 3853 practices (173 Comprehensive Primary Care Plus [CPC+] and 3680 non-CPC+). The trimmed sample (ie, final study sample) had a total of 469 practices (152 CPC+ and 317 non-CPC+).

Table 3 shows the difference-in-difference results for all study outcomes for patients diagnosed with depression or anxiety separately from those diagnosed with an OUD. Patients who were attributed to CPC+ practices and had a diagnosis of depression or anxiety filled more prescriptions for antidepressants, but this difference was not statistically significant (0.047 prescriptions filled per quarter [95% CI, −0.005 to 0.098]; *P* = .07). These patients filled statistically significantly more prescriptions for buprenorphine (0.024 prescriptions filled per quarter [95% CI, 0.006 to 0.041]; *P* = .007). These results are equivalent to 47 antidepressant prescriptions filled per 1000 patients per quarter and 24 buprenorphine prescriptions filled per 1000 patients per quarter. Patients diagnosed with depression or anxiety also had more visits to a community mental health center in 2021 (0.002 visits per quarter [95% CI, 0.0001 to 0.003]; *P* = .03) (Table 4). The relatively larger number of antidepressant prescriptions filled by these patients was observed only in 2021, while the relatively larger number of buprenorphine prescriptions was sustained in 2021 and 2022 (Table 4).

**Table 3.** Association Between CPC+ Participation and Spending, Health Care Utilization, and Prescription Fills Among Patients Diagnosed with Depression, Anxiety, or Opioid Use Disorder^a^

Outcome	Difference-in-differences, CPC+ vs non-CPC+, marginal effect (95% CI)	
	Members diagnosed with depression or anxiety^b^	Members diagnosed with opioid use disorder^c^
Total spending, $/patient/quarter	82 (−930 to 1095)	−623 (−2904 to 1658)
Health care utilization by site of care, No. of visits/patient/quarter		
PCP evaluation and management visits	0.041 (−0.032 to 0.115)	0.006 (−0.163 to 0.176)
Psychiatric hospitalizations	0.013 (−0.008 to 0.035)	0.145 (−0.137 to 0.428)
Community mental health center visits	0.001 (−0.001 to 0.003)	−0.003 (−0.008 to 0.003)
Substance use treatment (residential and nonresidential)	−0.009 (−0.052 to 0.034)	0.160 (−0.002 to 0.322)^d^
No. of prescriptions/patient/quarter		
Antidepressants	0.047 (−0.005 to 0.098)^d^	0.038 (−0.197 to 0.272)
Anxiolytics	−0.0003 (−0.035 to 0.035)	0.162 (0.005 to 0.319)^e^
Buprenorphine	0.024 (0.006 to 0.041)^f^	0.117 (0.037 to 0.196)^f^
Methadone	0.002 (−0.001 to 0.004)	0.001 (−0.020 to 0.022)
Naltrexone	0.004 (−0.002 to 0.011)	0.026 (−0.023 to 0.075)

Abbreviations: CPC+, Comprehensive Primary Care Plus; PCP, primary care physician.

^a^ Data are inverse probability score weighted.

^b^ The sample limited to patients diagnosed with depression or anxiety had a total of 1 846 043 patient-quarters (1 011 018 patient-quarters in CPC+ and 835 025 patient-quarters in non-CPC+). There were 101 550 unique patients attributed to CPC+ practices and 84 439 unique patients attributed to non-CPC+ practices.

^c^ The sample limited to patients diagnosed with an opioid use disorder had a total of 118 132 patient-quarters (53 554 patient-quarters in CPC+ and 64 578 patient-quarters in non-CPC+ practices). There were 4583 unique patients attributed to CPC+ practices and 5511 unique patients attributed to non-CPC+ practices.

^d^ *P* < .10.

^e^ *P* < .05.

^f^ *P* < .01.

**Table 4.** Association Between CPC+ Participation and Spending, Health Care Utilization, and Prescriptions Among Patients Diagnosed With Depression or Anxiety: Year by Year^a^

	Difference-in-differences, CPC+ vs non-CPC+, marginal effect (95% CI)		
	2020	2021	2022
Total spending, $/patient/quarter	−238 (−1403 to 928)	336 (−666 to 1337)	−99 (−1527 to 1329)
Site of care, No. of visits/patient/quarter			
PCP evaluation and management visits	0.042 (−0.042 to 0.126)	0.042 (−0.028 to 0.114)	0.014 (−0.077 to 0.105)
Psychiatric hospitalization	0.024 (−0.009 to 0.056)	0.013 (−0.006 to 0.032)	−0.012 (−0.037 to 0.013)
Community mental health center	−0.001 (−0.005 to 0.002)	0.002 (0.0001 to 0.003)^b^	0.002 (−0.001 to 0.004)
Substance use treatment (residential and nonresidential)	−0.008 (−0.041 to 0.026)	−0.006 (−0.051 to 0.039)	0.007 (−0.022 to 0.037)
No. of prescriptions/patient/quarter			
Antidepressants	0.017 (−0.030 to 0.064)	0.072 (0.0001 to 0.143)^b^	0.046 (−0.031 to 0.123)
Anxiolytics	−0.018 (−0.052 to 0.015)	−0.009 (−0.050 to 0.031)	0.022 (−0.057 to 0.100)
Buprenorphine	0.005 (−0.003 to 0.013)	0.009 (−0.002 to 0.020)^c^	0.066 (0.004 to 0.129)^b^
Methadone	0.001 (−0.002 to 0.003)	0.002 (−0.001 to 0.005)	0.001 (−0.003 to 0.006)
Naltrexone	0.008 (−0.006 to 0.022)	0.002 (−0.003 to 0.008)	0.001 (−0.005 to 0.006)

Abbreviations: CPC+, Comprehensive Primary Care Plus; PCP, primary care physician.

^a^ The sample limited to patients diagnosed with depression or anxiety had a total of 1 846 043 patient-quarters (1 011 018 patient-quarters from CPC+ practices and 835 025 patient-quarters from non-CPC+ practices). There were 101 550 unique patients attributed to CPC+ practices and 84 439 unique patients attributed to non-CPC+ practices. Data are inverse probability score weighted.

^b^ *P* < .05.

^c^ *P* < .10.

Patients with an OUD diagnosis who were attributed to CPC+ practices compared with non-CPC+ practices had numerically more visits for substance use treatment, but the results were not statistically significant (0.160 visits per quarter [95% CI, −0.002 to 0.322; *P* = .054), which is equivalent to 160 substance use treatment visits per 1000 patients per quarter. This difference was sustained in 2021 and 2022 (Table 5) and showed an increasing trend. In 2022, patients attributed to CPC+ practices filled more prescriptions for buprenorphine and fewer prescriptions for naltrexone. Patients attributed to CPC+ practices compared with non-CPC+ practices also filled more prescriptions for anxiolytics (0.162 prescriptions filled per quarter [95% CI, 0.005 to 0.319; *P* = .04) and buprenorphine (0.117 prescriptions filled per quarter [95% CI, 0.037 to 0.196]; *P* = .004) (Table 3). These results are equivalent to 162 anxiolytics prescriptions filled per 1000 patients per quarter, and 117 buprenorphine prescriptions filled per 1000 patients per quarter. This positive association in access to anxiolytics was sustained year over year since the start of the pandemic and showed an increasing trend. In 2022, patients attributed to CPC+ practices filled more prescriptions for buprenorphine and less prescriptions for naltrexone.

**Table 5.** Association Between CPC+ Participation and Spending, Health Care Utilization, and Prescriptions Among Patients Diagnosed With Opioid Use Disorder: Year by Year^a^

	Difference-in-differences between CPC+ vs non-CPC+, marginal effect (95% CI)		
	2020	2021	2022
Total spending, $/patient/quarter	−935 (−3177 to 1307)	942 (−2060 to 39440)	−2480 (−6069 to 1110)
Site of care, No. of visits/patient/quarter			
PCP evaluation and management visits	0.057 (−0.216 to 0.331)	0.049 (−0.131 to 0.229)	−0.178 (−0.469 to 0.114)
Psychiatric hospitalization	0.479 (−0.409 to 1.366)	−0.004 (−0.025 to 0.017)	−0.028 (−0.064 to 0.007)
Community mental health center	−0.013 (−0.032 to 0.007)	−0.001 (−0.005 to 0.004)	0.001 (−0.004 to 0.005)
Substance use treatment (residential and nonresidential)	0.048 (−0.172 to 0.268)	0.196 (0.036 to 0.357)^b^	0.219 (0.017 to 0.421)^b^
No. of prescriptions/patient/quarter			
Antidepressants	−0.098 (−0.321 to 0.125)	0.068 (−0.200 to 0.335)	0.066 (−0.267 to 0.400)
Anxiolytics	0.084 (−0.011 to 0.179)^c^	0.164 (−0.023 to 0.352)^c^	0.238 (−0.046 to 0.523)^c^
Buprenorphine	0.030 (−0.034 to 0.094)	0.042 (−0.031 to 0.115)	0.310 (0.089 to 0.531)^d^
Methadone	0.001 (−0.021 to 0.024)	−0.002 (−0.025 to 0.020)	−0.001 (−0.029 to 0.026)
Naltrexone	0.076 (−0.038 to 0.190)	0.006 (−0.020 to 0.032)	−0.017 (−0.030 to −0.004)^d^

Abbreviations: CPC+, Comprehensive Primary Care Plus; PCP, primary care physician.

^a^ The sample limited to patients diagnosed with an opioid use disorder had a total of 118 132 patient-quarters (53 554 patient-quarters from CPC+ practices and 64 578 patient-quarters from non-CPC+ practices). There were 4583 unique patients attributed to CPC+ practices and 5511 unique patients attributed to non-CPC+ practices. Data are inverse probability score weighted.

^b^ *P* < .05.

^c^ *P* < .10.

^d^ *P* < .01.

To check the robustness of the results, we estimated the models on a sample limited to patients who were continuously enrolled from 2018 to 2022. Results were similar to those of the main results. All analyses satisfied the pretreatment parallel trends assumption (eTables 7 and 8 in Supplement 1).

## Discussion

In this cohort study, findings were mixed on the association between CPC+ participation and use of mental health and substance use services. Study findings suggest that CPC+ was associated with moderately higher utilization of mental health services and substance use treatment for patients diagnosed with OUD, depression, or anxiety in the period after COVID-19 was declared a PHE. However, CPC+ participation was not associated with any of the other outcomes examined. Patients diagnosed with an OUD and attributed to a CPC+ practice filled more prescriptions for buprenorphine and anxiolytics and had higher use of substance use treatment services. Despite evidence of higher health care utilization, there was no difference in total costs between patients attributed to CPC+ and non-CPC+ practices.

These findings are particularly relevant in the context of the pandemic’s persistent toll on mental health and substance use morbidity and mortality.^1,15,16,17^ Drug overdose mortality continued to increase in 2022 with approximately 110 000 drug overdose deaths according to the Centers for Disease Control and Prevention.^18^ The CPC+ demonstration showed a consistent positive association with the utilization of medications for OUD and other substance use treatment for patients diagnosed with an OUD. The larger number of buprenorphine prescription fills among this group may have translated into lower morbidity and mortality due to buprenorphine’s potential to reduce the risk of all-cause and overdose-related mortality for these individuals.^19,20^

We also observed that the CPC+ demonstration was positively associated with prescription fills for anxiolytics among patients diagnosed with an OUD. One study reported on the high rates of co-occurrence of OUD and anxiety disorders, and their negative impact on the course of medication-assisted therapy. Individuals with co-occurring OUD and anxiety are more likely to have a relapse or discontinue treatment prematurely^21^; therefore, simultaneously treating these 2 conditions may improve outcomes for this population.^21^

The CPC+ practices were required to integrate behavioral health, including regular screening for mental health and substance use symptoms, which may have translated into better identification of patients needing mental health services and substance use treatment. Additionally, these practices used behavioral health specialists who might have been instrumental in helping patients navigate the complexities of mental health and substance use treatment.^22^

Importantly, the CPC+ demonstration took place in a continuum of advanced primary care initiatives. Several CPC+ practices participated in CPC Classic, which ran from 2012 to 2016, and several participated in other primary care transformation efforts.^8^ Previous participation in advanced primary care initiatives highlights the importance of keeping a long-term perspective on the benefits of advanced primary care demonstrations.^22^ Many CMMI demonstrations last 5 years, which may not be a long enough horizon to fully realize their potential in improving health care access, quality, and cost.^22^ An expert panel convened by the National Academies of Sciences, Engineering, and Medicine suggested that primary care initiatives have been wrongly evaluated based on short-term savings rather than on promoting high-quality primary care.^22^ Findings of the present study show that patients attributed to CPC+ practices had numerically (although not statistically significantly) higher utilization of substance use treatment even though CPC+ did not specifically require practices to improve their capabilities in this area.

The CPC+ demonstration likely yielded a greater return on investment for private payers due to their lower overall investment compared with CMS. In the fourth year of implementation, CMS provided 96% of funding for CPC+, with the remainder coming from private payers.^8^ Additionally, patients with other types of insurance coverage may have benefited from the CPC+ demonstration. Of the 15.3 million patients in the fourth year of CPC+, 10 million were uninsured or insured by nonpartnering payers.^8^

### Limitations

This study has several limitations. First, the findings are limited to Pennsylvania and while the sample size is large, we are careful not to generalize the findings to other regions. Second, we used a nonexperimental study design and while we strived to address selection bias, it is possible that our estimates are biased because CPC+ practices are inherently different from their nonparticipating counterparts. Third, several CPC+ practices had previously participated or were actively participating in other primary care transformation efforts, and our findings could have picked up spillover effects from other initiatives.

## Conclusions

Primary care has been the de facto mental health system for many individuals in the US.^23^ Primary care physicians are well positioned to address the increased demand for mental health and substance use services, but they need additional financial and technical support, perhaps through broader diffusion and adoption of effective models of care.^1,4,16^ This position is supported by the National Academies of Sciences, Engineering, and Medicine committee on high-quality primary care, which called for a reduction of financial and organizational barriers for expanding the role of primary care in providing mental health services.^22^ In this cohort study, patients with OUD who received care in CPC+ practices had more buprenorphine and anxiolytics prescription fills than patients with OUD who were attributed to non-CPC+ practices. Based on these findings, we believe that the CPC+ demonstration is a step in the right direction even with its modest support for behavioral health integration into primary care.^8^ The CMMI continues to invest in advanced primary care models that emphasize behavioral health integration, including Primary Care First and Making Care Primary. Understanding whether these models are associated with broader indicators of high-quality primary care is critical.

## Notes

Supplement 1. **eTable 1.** Healthcare Common Procedure Coding System (HCPCS) and Current Procedural Terminology (CPT) Codes for Evaluation and Management (E&M) Visits **eTable 2.** National Drug Codes (NDCs) for Buprenorphine From the US Food & Drug Administration NDC Directory **eTable 3.** National Drug Codes (NDCs) for Naltrexone From the US Food & Drug Administration NDC Directory **eTable 4.** National Drug Codes (NDCs) for Methadone From the US Food & Drug Administration NDC Directory **eTable 5.** National Drug Codes (NDCs) for Antidepressants From the US Food & Drug Administration NDC Directory **eTable 6.** National Drug Codes (NDCs) for Anxiolytics and Sedative-Hypnotics From the US Food & Drug Administration NDC Directory **eMethods**. Inverse Probability Score Weighting Approach: Probit Regression Results and Distribution of Propensity Scores Across Study Groups **eTable 7.** Baseline Trends Test (Parallel Trends Test): Primary Study Outcomes for Patients Diagnosed With Anxiety or Depression **eTable 8.** Baseline Trends Test (Parallel Trends Test): Primary Study Outcomes for Patients Diagnosed With Opioid Use Disorder

Supplement 2.
Data Sharing Statement
