# Supplementary material for: Access to Mental Health and Substance Use Treatment in Comprehensive Primary Care Plus
Source: JAMA Netw Open. 2024 Apr 26;7(4):e248519. doi: 10.1001/jamanetworkopen.2024.8519 (PMC11053373; doi:10.1001/jamanetworkopen.2024.8519)
Supplement: Supplement 1. — eTable 1. Healthcare Common Procedure Coding System (HCPCS) and Current Procedural Terminology (CPT) Codes for Evaluation and Management (E&M) Visits eTable 2. National Drug Codes (NDCs) for Buprenorphine From the US Food & Drug Administration NDC Directory eTable 3. National Drug Codes (NDCs) for Naltrexone From the US Food & Drug Administration NDC Directory eTable 4. National Drug Codes (NDCs) for Methadone From the US Food & Drug Administration NDC Directory eTable 5. National Drug Codes (NDCs) for Antidepressants From the US Food & Drug Administration NDC Directory eTable 6. National Drug Codes (NDCs) for Anxiolytics and Sedative-Hypnotics From the US Food & Drug Administration NDC Directory eMethods. Inverse Probability Score Weighting Approach: Probit Regression Results and Distribution of Propensity Scores Across Study Groups eTable 7. Baseline Trends Test (Parallel Trends Test): Primary Study Outcomes for Patients Diagnosed With Anxiety or Depression eTable 8. Baseline Trends Test (Parallel Trends Test): Primary Study Outcomes for Patients Diagnosed With Opioid Use Disorder [file jamanetwopen-e248519-s001.pdf]

# Supplemental Online Content

Santos T, Bergman A, Smith-McLallen A. Access to mental health and substance use treatment in Comprehensive Primary Care Plus. *JAMA Network Open*. 2024;7(4):e248519. doi:10.1001/jamanetworkopen.2024.8519

**eTable 1.** Healthcare Common Procedure Coding System (HCPCS) and Current Procedural Terminology (CPT) Codes for Evaluation and Management (E&M) Visits

**eTable 2.** National Drug Codes (NDCs) for Buprenorphine From the US Food & Drug Administration NDC Directory

**eTable 3.** National Drug Codes (NDCs) for Naltrexone From the US Food & Drug Administration NDC Directory

**eTable 4.** National Drug Codes (NDCs) for Methadone From the US Food & Drug Administration NDC Directory

**eTable 5.** National Drug Codes (NDCs) for Antidepressants From the US Food & Drug Administration NDC Directory

**eTable 6.** National Drug Codes (NDCs) for Anxiolytics and Sedative-Hypnotics From the US Food & Drug Administration NDC Directory

**eMethods.** Inverse Probability Score Weighting Approach: Probit Regression Results and Distribution of Propensity Scores Across Study Groups

**eTable 7.** Baseline Trends Test (Parallel Trends Test): Primary Study Outcomes for Patients Diagnosed With Anxiety or Depression

**eTable 8.** Baseline Trends Test (Parallel Trends Test): Primary Study Outcomes for Patients Diagnosed With Opioid Use Disorder

This supplemental material has been provided by the authors to give readers additional information about their work.

**eTable 1. Healthcare Common Procedure Coding System (HCPCS) and Current Procedural Terminology (CPT) Codes for Evaluation and Management (E&M) Visits**

| **Evaluation and Management** (E&M) | **Description** |
|---|---|
| 99201 | Office/Outpatient Services |
| 99202 | Office/Outpatient Services |
| 99203 | Office/Outpatient Services |
| 99204 | Office/Outpatient Services |
| 99205 | Office/Outpatient Services |
| 99211 | Office/Outpatient Services |
| 99212 | Office/Outpatient Services |
| 99213 | Office/Outpatient Services |
| 99214 | Office/Outpatient Services |
| 99215 | Office/Outpatient Services |
| 99241 | Outpatient Consultations |
| 99242 | Outpatient Consultations |
| 99243 | Outpatient Consultations |
| 99244 | Outpatient Consultations |
| 99245 | Outpatient Consultations |
| 99304 | Nursing Facility Services |
| 99305 | Nursing Facility Services |
| 99306 | Nursing Facility Services |
| 99307 | Nursing Facility Services |
| 99308 | Nursing Facility Services |
| 99309 | Nursing Facility Services |
| 99310 | Nursing Facility Services |
| 99315 | Nursing Facility Services |
| 99316 | Nursing Facility Services |
| 99318 | Nursing Facility Services |
| 99324 | Domiciliary, Rest Home or Custodial Care Services |
| 99325 | Domiciliary, Rest Home or Custodial Care Services |
| 99326 | Domiciliary, Rest Home or Custodial Care Services |
| 99327 | Domiciliary, Rest Home or Custodial Care Services |
| 99328 | Domiciliary, Rest Home or Custodial Care Services |
| 99334 | Domiciliary, Rest Home or Custodial Care Services |
| 99335 | Domiciliary, Rest Home or Custodial Care Services |
| 99336 | Domiciliary, Rest Home or Custodial Care Services |
| 99337 | Domiciliary, Rest Home or Custodial Care Services |
| 99339 | Domiciliary, Rest Home or Home Care Plan Oversight Services |
| 99340 | Domiciliary, Rest Home or Home Care Plan Oversight Services |
| 99341 | Home Services |
| 99342 | Home Services |
| 99343 | Home Services |

**eTable 1 (continued). Healthcare Common Procedure Coding System (HCPCS) and Current Procedural Terminology (CPT) Codes for Evaluation and Management (E&M) Visits**

| Evaluation and Management (E&M) | Description |
|---|---|
| 99344 | Home Services |
| 99345 | Home Services |
| 99347 | Home Services |
| 99348 | Home Services |
| 99349 | Home Services |
| 99350 | Home Services |
| 99374 | Care Plan Oversight Services |
| 99375 | Care Plan Oversight Services |
| 99377 | Care Plan Oversight Services |
| 99378 | Care Plan Oversight Services |
| 99379 | Care Plan Oversight Services |
| 99380 | Care Plan Oversight Services |
| 99381 | Preventive Medicine Services |
| 99382 | Preventive Medicine Services |
| 99383 | Preventive Medicine Services |
| 99384 | Preventive Medicine Services |
| 99385 | Preventive Medicine Services |
| 99386 | Preventive Medicine Services |
| 99387 | Preventive Medicine Services |
| 99391 | Preventive Medicine Services |
| 99392 | Preventive Medicine Services |
| 99393 | Preventive Medicine Services |
| 99394 | Preventive Medicine Services |
| 99395 | Preventive Medicine Services |
| 99396 | Preventive Medicine Services |
| 99397 | Preventive Medicine Services |
| 99401 | Preventive Medicine Services |
| 99402 | Preventive Medicine Services |
| 99403 | Preventive Medicine Services |
| 99404 | Preventive Medicine Services |
| 99406 | New or Established Patient Behavior Change Interventions, Individual |
| 99407 | New or Established Patient Behavior Change Interventions, Individual |
| 99408 | New or Established Patient Behavior Change Interventions, Individual |
| 99409 | New or Established Patient Behavior Change Interventions, Individual |
| 99411 | New or Established Patient Preventive Medicine, Group Counseling |
| 99412 | New or Established Patient Preventive Medicine, Group Counseling |
| G0402 | Medicare Preventive Services |
| G0438 | Medicare Preventive Services |
| G0439 | Medicare Preventive Services |
| 99495 | TCM 14-days |
| 99496 | TCM 7-days |

**eTable 2. National Drug Codes (NDCs) for Buprenorphine From the US Food & Drug Administration NDC Directory**

| NDC (Buprenorphine) | | | | |
|---|---|---|---|---|
| 54017613 | 12496010005 | 43598058201 | 54123091430 | 62756096983 |
| 54017713 | 12496030001 | 43598058230 | 54123092930 | 62756097083 |
| 54018813 | 12496030002 | 47781035503 | 54123095730 | 63629402801 |
| 54018913 | 12496030005 | 47781035511 | 54123098630 | 63629403401 |
| 93537856 | 12496120201 | 47781035603 | 54569549600 | 63629403402 |
| 93537956 | 12496120801 | 47781035611 | 54569573900 | 63629403403 |
| 93572056 | 12496120803 | 47781035703 | 54569573901 | 63629409201 |
| 93572156 | 12496121201 | 47781035711 | 54569573902 | 63874108403 |
| 228315303 | 12496121203 | 47781035803 | 54569639900 | 63874108503 |
| 228315403 | 12496127802 | 47781035811 | 54569640800 | 63874117303 |
| 228315473 | 12496128302 | 49999039507 | 54569657800 | 65162041503 |
| 228315503 | 12496130602 | 49999039515 | 54868570700 | 65162041603 |
| 228315567 | 12496131002 | 49999039530 | 54868570701 | 66336001630 |
| 228315573 | 16590066605 | 49999063830 | 54868570702 | 68071138003 |
| 228315603 | 16590066630 | 49999063930 | 54868570703 | 68071151003 |
| 378092393 | 16590066705 | 50090292400 | 54868570704 | 68258299103 |
| 378092493 | 16590066730 | 50268014411 | 54868575000 | 68258299903 |
| 378876716 | 16590066790 | 50268014415 | 55045378403 | 68308020230 |
| 378876793 | 23490927003 | 50268014511 | 55700014730 | 68308020830 |
| 378876816 | 23490927006 | 50268014515 | 55700018430 | 5938502160 |
| 378876893 | 23490927009 | 50383028793 | 55700030230 | 5938502260 |
| 406192303 | 35356000407 | 50383029493 | 55700030330 | 5938502360 |
| 406192403 | 35356000430 | 50383092493 | 58284010014 | 5938502460 |
| 406802003 | 35356055530 | 50383093093 | 59385001201 | 5938502560 |
| 406800503 | 35356055630 | 52427069203 | 59385001230 | 5938502660 |
| 490005100 | 42291017430 | 52427069211 | 59385001401 | 5938502760 |
| 490005130 | 42291017530 | 52427069403 | 59385001430 | 5570086760 |
| 490005160 | 42858050103 | 52427069411 | 59385001601 | 5938501430 |
| 490005190 | 42858050203 | 52427069803 | 59385001630 | 7133503536 |
| 781721606 | 43063018407 | 52427069811 | 60429058611 | 7133509502 |
| 781721664 | 43063018430 | 52427071203 | 60429058630 | 4285858640 |
| 781722706 | 43063066706 | 52427071211 | 60429058633 | 7133509504 |
| 781722764 | 43063075306 | 52440010014 | 60429058711 | 6275645983 |
| 781723806 | 43598057901 | 52959030430 | 60429058730 | 7133503533 |
| 781723864 | 43598057930 | 52959074930 | 60429058733 | 6275646083 |
| 781724906 | 43598058001 | 53217013830 | 62175045232 | 7133503535 |
| 781724964 | 43598058030 | 53217024630 | 62175045832 | 7133503534 |
| 12496010001 | 43598058101 | 54123011430 | 62756045983 | 7133503532 |
| 12496010002 | 43598058130 | 54123090730 | 62756046083 | 228315303 |
| 228315603 | 93365840 | 7051820141 | 6362971265 | 7133515141 |
| 4285850103 | 228315403 | 54017613 | 7133518589 | 6516241509 |
| 5570057904 | 228315503 | 54017713 | 7133516531 | 7133515142 |

**eTable 2 (continued). National Drug Codes (NDCs) for Buprenorphine, Naltrexone, and Methadone From the US Food & Drug Administration NDC Directory**

| NDC (Buprenorphine) | | | | |
|---|---|---|---|---|
| 93365740 | 4359858030 | 7051822260 | 7133517201 | 143924605 |
| 7133503531 | 378876593 | 7051822261 | 7133517202 | 6362971263 |
| 7133509505 | 378876693 | 7051822262 | 7133517251 | 93360121 |
| 7133509507 | 6362972701 | 7051822263 | 1672954910 | 93365721 |
| 6275645964 | 6362972702 | 5811801778 | 1672955010 | 47781047574 |
| 6275646064 | 4359857930 | 5811801768 | 904700906 | 69238120502 |
| 7133509506 | 4285860103 | 7133511631 | 5570090130 | 51552076502 |
| 6068748121 | 4285860203 | 7133511632 | 7133518580 | 47781047474 |
| 6068749221 | 6275697064 | 7133511633 | 7133518581 | 42858083940 |
| 4285835340 | 4778135503 | 7133511634 | 904701006 | 42858035340 |
| 6704699430 | 4778135603 | 7133511635 | 7133518582 | 42858049340 |
| 6704699530 | 4778135703 | 7133511636 | 7133518583 | 69238120302 |
| 7133503537 | 4778135803 | 7133511637 | 7133518584 | 93360240 |
| 5009058050 | 378876893 | 7133511638 | 7133518585 | 42858060203 |
| 4285875040 | 5038329493 | 7133511639 | 7133518586 | 69238120202 |
| 93365940 | 6275696983 | 5570030230 | 7133518587 | 62991158301 |
| 4285849340 | 5242769203 | 6516241503 | 7133518588 | 60687049221 |
| 4285850203 | 5242769403 | 5026814415 | 6704699730 | 16729055010 |
| 4285883940 | 7051823110 | 7051810070 | 6704699630 | 378876516 |
| 7133509501 | 5242769803 | 5026814515 | 5901175704 | 42858058640 |
| 6923812022 | 5242771203 | 6516241609 | 5901175004 | 93360140 |
| 6923812032 | 6042958730 | 6362972691 | 5901175204 | 47781047274 |
| 6923812042 | 378876793 | 6516241603 | 5901175804 | 93360040 |
| 6923812052 | 5038328793 | 6362971264 | 5901175104 | 51927101200 |
| 6923815052 | 4359858130 | 6362971266 | 1249601001 | 42858075040 |
| 93365640 | 6217545832 | 6362971269 | 1249603001 | 47781040074 |
| 7133509503 | 6042958630 | 6362971261 | 1249612023 | 69238150502 |
| 7133511541 | 7051831290 | 6362971267 | 1249612043 | 47781047374 |
| 7133511542 | 6275696964 | 6362971268 | 1249612083 | 47781042574 |
| 7133511543 | 4359858230 | 4202317905 | 1249612123 | 69238120402 |
| 7133511544 | 7133512961 | 517072505 | 5412311430 | 93360340 |
| 7133511545 | 7133512962 | 5038392493 | 5412390730 | 59011075004 |
| 7133511546 | 6275697083 | 6362971262 | 5412391430 | 59011075104 |
| 7133511547 | 6217545232 | 5009015710 | 5412392930 | 59011075204 |
| 7133511548 | 7133513781 | 5038393093 | 5412395730 | 59011075704 |
| 7133511549 | 7051820140 | 409201232 | 5412398630 | 59011075804 |

| Brand and Generic Names (Buprenorphine) | | |
|---|---|---|
| Bunavail | Buprenorphine HCl-Naloxone HCl | Sublocade |
| Buprenorphine | Bunavail | Suboxone |
| Buprenorphine HCl | Butrans | Zubsolv |

**eTable 3. National Drug Codes (NDCs) for Naltrexone From the US Food & Drug Administration NDC Directory**

| NDC (Naltrexone) | | |
|---|---|---|
| 56001122 | 16729008101 | 52152010504 |
| 56001130 | 16729008110 | 52152010530 |
| 56001170 | 42291063230 | 54868557400 |
| 56007950 | 43063059115 | 63459030042 |
| 56008050 | 47335032683 | 65694010003 |
| 185003901 | 47335032688 | 65694010010 |
| 185003930 | 50436010501 | 65757030001 |
| 406009201 | 51224020630 | 65757030202 |
| 406009203 | 51224020650 | 68084029111 |
| 406117001 | 51285027501 | 68084029121 |
| 406117003 | 51285027502 | 68094085362 |
| 555090201 | 52152010502 | 68115068030 |
| 555090202 | | |

| Brand and Generic Names (Naltrexone) |
|---|
| Naltrexone |
| Naltrexone HCl |
| Naltrexone HCl Dihydrate |
| Vivitrol |

**eTable 4. National Drug Codes (NDCs) for Methadone From the US Food & Drug Administration NDC Directory**

| NDC (Methadone) | | |
|---|---|---|
| 54355344 | 406577101 | 406575501 |
| 54039168 | 13107008901 | 42806031701 |
| 66689069430 | 31722094701 | 31722094601 |
| 66689069479 | 67877011601 | 54070925 |
| 54039268 | 54071020 | 54457025 |
| 54355663 | 54071025 | 13107008801 |
| 54355563 | 42806031801 | 68462080001 |
| 66689081010 | 904653061 | 406575562 |
| 54457125 | 68462080101 | |

| Brand and Generic Names (Methadone) |
|---|
| Methadone HCl |
| Methadone HCl Intensol |

**eTable 5. National Drug Codes (NDCs) for Antidepressants From the US Food & Drug Administration NDC Directory**

***Note:*** NDCs available upon request. There were 1,774 unique NDC codes.

| Brand and Generic Names (Antidepressant) | | |
|---|---|---|
| **SSRI** | **SNRI** | **Tricyclic** |
| Citalopram | Desvenlafaxine | Amitriptyline |
| Escitalopram | Duloxetine | Amoxapine |
| Fluoxetine | Levomilnacipran | Clomipramine |
| Fluvoxamine | Venlafaxine | Desipramine |
| Paroxetine | Milnacipran | Doxepin |
| Sertraline | Desvenlafaxine er | Imipramine |
| Vilazodone | Desvenlafaxine fumarate er | Maprotiline |
| Citalopram hydrobromide | Desvenlafaxine succinate er | Nortriptyline |
| Celexa | Pristiq | Protriptyline |
| Escitalopram oxalate | Khedezla | Trimipramine |
| Lexapro | Duloxetine HCl | Mirtazapine |
| Fluoxetine HCl | Cymbalta | Amitriptyline HCl |
| Fluoxetine HCl (PMDD) | Fetzima | Chlordiazepoxide-Amitriptyline |
| Olanzapine-fluoxetine HCl | Fetzima Titration | Perphenazine-Amitriptyline |
| Prozac | Effexor XR | Elavil |
| Prozac Weekly | **Tetracyclic** | Amoxapine |
| Sarafem | Maprotiline HCl | Clomipramine HCl |
| Symbyax | Mirtazapine | Clomipramine HCl |
| Fluvoxamine Maleate | Remeron | Anafranil |
| Fluvoxamine Maleate ER | Remeron Soltab | Desipramine HCl |
| Fluvoxamine Maleate | | Doxepin HCl |
| Paroxetine HCl | | Imipramine HCl |
| Paroxetine HCl ER | | Imipramine Pamoate |
| Paroxetine Mesylate | | Tofranil |
| Paxil | | Maprotiline HCl |
| Paxil CR | | Nortriptyline HCl |
| Brisdelle | | Pamelor |
| Pexeva | | Protriptyline HCl |
| Sertraline HCl | | Trimipramine Maleate |
| Zoloft | | Surmontil |
| | | Mirtazapine |
| | | Remeron |
| | | Remeron soltab |

**eTable 6. National Drug Codes (NDCs) for Anxiolytics and Sedative-Hypnotics From the US Food & Drug Administration NDC Directory**

***Note:*** NDCs available upon request. There were 1,221 unique NDC codes.

| Brand and Generic Names (Anxiolytic and Sedative-Hypnotics) | |
|---|---|
| Alprazolam | Midazolam HCl (pf) |
| Chlordiazepoxide | Tranxene-t |
| Clorazepate | Xanax XR |
| Diazepam | Buspirone HCl |
| Estazolam | Eszopiclone |
| Flurazepam | Lunesta |
| Halazepam | Hydroxyzine HCl |
| Lorazepam | Hydroxyzine pamoate |
| Midazolam | Hydroxyzine HCl |
| Oxazepam | Hydroxyzine pamoate |
| Prazepam | Vistaril |
| Quazepam | Meprobamate |
| Temazepam | Promethazine HCl |
| Triazolam | Promethazine VC |
| Clobazam | Promethazine VC plain |
| Clonazepam | Promethazine VC/Codeine |
| Ativan | Promethazine-Codeine |
| Dalmane | Promethazine-DM |
| Klonopin | Promethazine-Phenyleph-Codeine |
| Halcion | Promethazine-Phenylephrine |
| Librium | Ramelteon |
| Restoril | Rozerem |
| Serax | Zaleplon |
| Tranxene | Zolpidem Tartrate |
| Valium | Zolpidem Tartrate ER |
| Xanax | Zolpimist |
| Azapirones | Ambien |
| Buspar | Ambien CR |
| Buspirone | Edluar |
| Chloral hydrate | Intermezzo |
| Alprazolam ER | RA Sleep Aid |
| Alprazolam Intensol | RA Sleep Aid (diphenhydramine) |
| Alprazolam XR | RA Nighttime Sleep Aid |
| Chlordiazepoxide HCl | |
| Chlordiazepoxide-Clidinium | |
| Diazepam Intensol | |
| Flurazepam HCl | |
| Lorazepam Intensol | |
| Midazolam HCl | |

**eMethods. Inverse Probability Score Weighting Approach: Probit Regression Results and Distribution of Propensity Scores Across Study Groups**

To enroll in CPC+, primary care practices had to meet certain requirements. Chiefly, practices were required to serve a minimum of 150 Medicare fee-for-service beneficiaries, pass a Centers for Medicare & Medicaid Services (CMS) program integrity screening, and have certified health information technology practices in place, including use of electronic health records and Electronic Clinical Quality Measures. Some practices that met these requirements still chose not to enroll in CPC+. As a result of both enrollment requirements and self-selection into the program, CPC+ practices can look inherently different from other practices across numerous dimensions.

A valid comparison of CPC+ practices to non-CPC+ practices must account for the baseline differences in practice characteristics, which may affect the outcomes examined in our study. In our analysis, we employed an Inverse Probability Weighting (IPW) methodology to balance our sample.

We first estimate the probability of a practice being a CPC+ practice using a probit regression:

$$1[CPC+_i] = \beta_0 + \beta_1 physicians_i + \beta_2 physicians^2 + \beta_3 members + \beta_4 members^2 + \beta_5 age + \beta_6 age^2 + \beta_7 male + \beta_8 male^2 + \beta_9 MA + \beta_{10} MA^2 + \beta_{11} HMO + \beta_{12} HMO^2 + \epsilon$$

Where we control for both the linear and quadratic terms of: (a) the number of physicians operating in the practice; (b) the number of patients attributed to the practice; (c) the average age of patients in the practice; (d) the share of male patients in the practice; (e) the share of Medicare Advantage patients in the practice; and (f) the share of HMO patients in the practice. Regression results are reported in eMethods Table 1. The model is moderately to highly successful in predicting CPC+ enrollment, with a model pseudo R-squared of 0.579.

We use the predicted probabilities of our probit model to construct inverse-probability weights. eMethods Table 2 reports on the covariate balance in the raw sample and in our sample following inverse-probability weighting. Columns (1) and (3) report the mean difference in the standardized variables in the raw sample and the inverse-probability weighted sample, respectively. Columns (2) and (4) report the p-value of the corresponding standardized difference for each sample. In the raw sample, we find a statistically significant difference in means across all reported practice characteristics. After weighting, only three practice characteristics exhibit a mean difference that is statistically significant at the 95% level: the percent of practice members with hypertension, diabetes, and anxiety disorders.

Finally, eMethods Figure 1 plots the predicted propensity score from our probit regression, restricted to our trimmed sample (omitting the bottom and top 5% of the sample in terms of inverse-probability weights). Compared to the raw sample, the distribution of CPC+ practices and non-CPC+ practices track more closely in the inverse-probability weighted sample.

**IPW Probit Regression Results**

| Variable | Coefficient |
|---|---|
| Number of physicians | 0.0271** |
| | (0.0135) |
| Number of physicians, squared | -0.000249 |
| | (0.000167) |
| Number of members | 0.00114*** |
| | (9.27e-05) |
| Number of members, squared | -8.41e-08*** |
| | (1.29e-08) |
| Average age | 0.129*** |
| | (0.0336) |
| Average age, squared | -0.000831** |
| | (0.000396) |
| Percent male | 2.415 |
| | (2.555) |
| Percent male, squared | -5.320* |
| | (3.195) |
| Percent MA | 3.455*** |
| | (1.145) |
| Percent MA, squared | -2.266* |
| | (1.335) |
| Percent HMO | 6.001*** |
| | (2.106) |
| Percent HMO, squared | -5.880*** |
| | (1.932) |
| Constant | -8.482*** |
| | (1.103) |
| Observations | 3,926 |
| Pseudo R-squared | 0.579 |

Notes: Robust standard errors in parentheses, *** p<0.01, ** p<0.05, * p<0.1

**Covariate Balance Following Inverse-Probability Weighting**

| Practice characteristic | Sample before IPW (n=469 practices) Standardized difference | Sample before IPW (n=469 practices) P-value of difference | Sample after IPW (n=469 practices) Standardized difference | Sample after IPW (n=469 practices) P-value of difference |
|---|---|---|---|---|
| | *A* | *B* | *C* | *D* |
| ***Practice Size*** | | | | |
| Number of physicians | 0.46 | <0.001 | 0.03 | 0.72 |
| Number of attributed patients | 0.83 | <0.001 | -0.17 | 0.27 |
| ***Patient Demographics*** | | | | |
| Male (%) | -0.19 | 0.06 | -0.02 | 0.85 |
| Average age | -0.32 | <0.001 | 0.00 | 0.99 |
| Aged 65 and over (%) | -0.30 | 0.001 | 0.01 | 0.96 |
| ***Patient physical chronic conditions (%):*** | | | | |
| Hyperlipidemia | -0.62 | <0.001 | -0.22 | 0.12 |
| Hypertension | -0.67 | <0.001 | -0.32 | 0.02 |
| RA/Osteoarthritis | -0.52 | <0.001 | -0.13 | 0.51 |
| Diabetes | -0.62 | <0.001 | -0.33 | 0.002 |
| COPD | -0.27 | 0.004 | -0.02 | 0.88 |
| Ischemic Heart Disease | -0.48 | <0.001 | -0.18 | 0.14 |
| Chronic kidney disease | -0.42 | <0.001 | -0.06 | 0.72 |
| Heart Failure | -0.34 | <0.001 | -0.01 | 0.97 |
| ***Patient behavioral chronic conditions (%):*** | | | | |
| Anxiety disorders | 0.32 | <0.001 | 0.24 | 0.007 |
| Depressive disorders | 0.18 | 0.04 | 0.12 | 0.25 |
| Drug use disorders | -0.30 | <0.001 | -0.21 | 0.06 |
| Opioid use disorders | -0.29 | <0.001 | -0.17 | 0.08 |
| Alcohol use disorders | -0.25 | 0.001 | -0.12 | 0.28 |
| Bipolar disorders | 0.00 | 0.97 | 0.02 | 0.80 |
| Schizophrenia and other psychotic disorders | -0.17 | 0.06 | -0.07 | 0.42 |
| ***Patient insurance coverage (%):*** | | | | |
| PPO coverage | 0.44 | <0.001 | 0.23 | 0.11 |
| HMO coverage | -0.31 | <0.001 | -0.15 | 0.30 |

Abbreviations: IPW, inverse probability weighting; RA, rheumatoid arthritis; COPD, Chronic Obstructive Pulmonary Disease; PPO, preferred provider organization; HMO, health maintenance organization

Differences were calculated from standardized variables using baseline data from 2019, comparing the final study sample before and after inverse-probability weighting. To get the final study sample, the raw sample (i.e., all primary care practices) was trimmed by omitting the bottom and top 5% of the sample in terms of inverse-probability weights. The raw sample had a total of 3,853 practices (173 CPC+ and 3,680 non-CPC+). The trimmed sample (i.e., final study sample) had a total of 469 practices (152 CPC+ and 317 non-CPC+).

**Propensity Score Distribution Following Inverse-Probability Weighting**

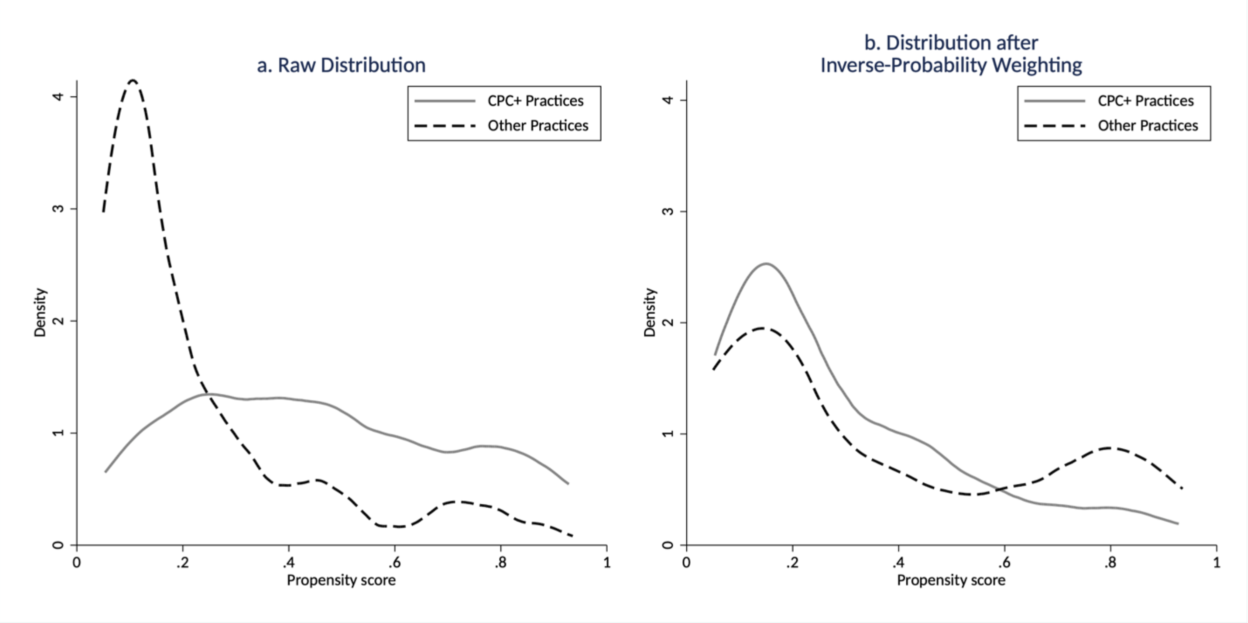

**eTable 7. Baseline Trends Test (Parallel Trends Test): Primary Study Outcomes for Patients Diagnosed With Anxiety or Depression**

| | Total Spending | PCP E&M | Psych Hosp | Comm MH Ctr Visits | Subst Use Treat | Antidep | Anxiolytic | Bupre | Methadone | Naltrexone |
|---|---|---|---|---|---|---|---|---|---|---|
| **treat*Q2.2018** | -1,217 | -0.0236 | -0.0164 | 0.000510 | -0.0589 | -0.0508 | -0.0394 | -0.00468 | 0.00204 | 0.00191 |
| | (1,384) | (0.0296) | (0.0123) | (0.00141) | (0.0486) | (0.0407) | (0.0337) | (0.00473) | (0.00153) | (0.00235) |
| **treat*Q3.2018** | -1,287 | -0.0329 | -0.00621 | 0.000487 | -0.111 | -0.0135 | -0.0608 | -0.00194 | 8.46e-05 | 0.00449 |
| | (1,250) | (0.0306) | (0.0108) | (0.00118) | (0.0890) | (0.0356) | (0.0341) | (0.00667) | (0.00139) | (0.00359) |
| **treat*Q4.2018** | -3,315 | -0.0400 | -0.0106 | 0.00101 | -0.112 | -0.0159 | **-0.0753*** | 0.000699 | 0.000160 | 0.00493 |
| | (1,856) | (0.0556) | (0.0274) | (0.00113) | (0.0949) | (0.0576) | (0.0383) | (0.00547) | (0.00114) | (0.00309) |
| **treat*Q1.2019** | -1,368 | -0.0446 | 0.0211 | 0.000688 | -0.0591 | -0.000614 | -0.0537 | **-0.0136*** | 0.00224 | 0.00267 |
| | (1,269) | (0.0481) | (0.0190) | (0.00116) | (0.0600) | (0.0463) | (0.0383) | (0.00572) | (0.00162) | (0.00357) |
| **treat*Q2.2019** | **-2,843**** | -0.112 | -0.0272 | 0.00118 | 0.246 | -0.0117 | -0.0587 | 0.00254 | 0.00482 | 0.00126 |
| | (1,080) | (0.0715) | (0.0246) | (0.00135) | (0.255) | (0.0408) | (0.0416) | (0.00548) | (0.00272) | (0.00371) |
| **treat*Q3.2019** | -2,032 | -0.0216 | -0.00391 | -0.000728 | -0.0916 | 0.0403 | -0.0629 | -0.00490 | 0.00328 | 0.00218 |
| | (1,509) | (0.0889) | (0.0219) | (0.00124) | (0.0881) | (0.0408) | (0.0334) | (0.00830) | (0.00214) | (0.00606) |
| **treat*Q4.2019** | 697.6 | -0.0754 | 0.0151 | -0.00207 | -0.0499 | -0.0178 | -0.0748 | -0.00330 | **0.00453**** | -0.00594 |
| | (1,542) | (0.0424) | (0.0200) | (0.00151) | (0.0523) | (0.0634) | (0.0456) | (0.00643) | (0.00167) | (0.00723) |

**** p<0.01, * p<0.05**

**Psych Hosp**: psychiatric hospitalization
**Comm MH Ctr Visits**: community mental health center visits
**Subst Use Treat**: substance use treatment
**Antidep:** antidepressant
**Bupre:** buprenorphine

We tested for pre-treatment parallel trends by limiting the sample to the pre-treatment period (2018 to 2019) and tested the significance of the interaction between the treatment variable and the linear quarter dummies (i.e., treat*Qx.YEAR). A non-significant p-value indicates that the pre-treatment trends are parallel.

**eTable 8. Baseline Trends Test (Parallel Trends Test): Primary Study Outcomes for Patients Diagnosed with Opioid Use Disorder**

| | Total Spending | PCP E&M | Psych Hosp | Comm MH Ctr Visits | Subst Use Treat | Antidep | Anxiolytic | Bupre | Methadone | Naltrexone |
|---|---|---|---|---|---|---|---|---|---|---|
| **treat*Q2.2018** | 3,190 | -0.0593 | -0.0722 | -0.00239 | -0.294 | 0.0401 | -0.221 | -0.0332 | 0.00822 | 0.00493 |
| | (3,850) | (0.0702) | (0.0452) | (0.00143) | (0.258) | (0.115) | (0.133) | (0.0291) | (0.0140) | (0.0120) |
| **treat*Q3.2018** | 2,403 | 0.0736 | -0.0301 | **-0.00260*** | -0.665 | -0.0667 | -0.128 | -0.0171 | -0.0162 | 0.00670 |
| | (3,959) | (0.115) | (0.0470) | (0.00120) | (0.554) | (0.0779) | (0.0966) | (0.0420) | (0.0126) | (0.00882) |
| **treat*Q4.2018** | -2,050 | 0.0892 | -0.0154 | -0.00189 | -0.243 | -0.0558 | -0.242 | 0.00250 | -0.0189 | 0.00833 |
| | (2,858) | (0.0725) | (0.0584) | (0.00160) | (0.405) | (0.166) | (0.147) | (0.0354) | (0.0107) | (0.0120) |
| **treat*Q1.2019** | -3,724 | **-0.254*** | -0.000345 | -0.00237 | -0.233 | 0.0357 | -0.134 | **-0.0941*** | -0.00447 | 0.00265 |
| | (2,849) | (0.128) | (0.0615) | (0.00205) | (0.339) | (0.0950) | (0.0762) | (0.0433) | (0.0134) | (0.0141) |
| **treat*Q2.2019** | -4,100 | -0.0208 | -0.212 | -0.000739 | -0.309 | 0.0254 | -0.168 | -0.00216 | 0.00786 | 0.00872 |
| | (3,427) | (0.0732) | (0.146) | (0.00202) | (0.328) | (0.124) | (0.116) | (0.0378) | (0.0241) | (0.0122) |
| **treat*Q3.2019** | **-7,341*** | -0.0274 | 0.0174 | -0.00123 | 0.0224 | 0.0573 | -0.127 | -0.0468 | -0.00658 | 0.0465 |
| | (3,515) | (0.264) | (0.129) | (0.00408) | (0.378) | (0.122) | (0.130) | (0.0626) | (0.0206) | (0.0338) |
| **treat*Q4.2019** | -1,086 | -0.0911 | 0.0750 | 0.000704 | -0.356 | -0.127 | -0.108 | -0.0353 | 0.00243 | -0.000925 |
| | (3,301) | (0.0865) | (0.101) | (0.00272) | (0.316) | (0.216) | (0.111) | (0.0463) | (0.0177) | (0.0143) |

**** p<0.01, * p<0.05**

**Psych Hosp**: psychiatric hospitalization
**Comm MH Ctr Visits**: community mental health center visits
**Subst Use Treat**: substance use treatment
**Antidep:** antidepressant
**Bupre:** buprenorphine

We tested for pre-treatment parallel trends by limiting the sample to the pre-treatment period (2018 to 2019) and tested the significance of the interaction between the treatment variable and the linear quarter dummies (i.e., treat*Qx.YEAR). A non-significant p-value indicates that the pre-treatment trends are parallel.
