# Supplementary material for: Access to Mental Health and Substance Use Treatment in Comprehensive Primary Care Plus
Source: JAMA Netw Open. 2024 Apr 26;7(4):e248519. doi: 10.1001/jamanetworkopen.2024.8519 (PMC11053373; doi:10.1001/jamanetworkopen.2024.8519)
Supplement: Supplement 2. — Data Sharing Statement [file jamanetwopen-e248519-s002.pdf]

Data Sharing Statement

Santos. Access to Mental Health and Substance Use Treatment in Comprehensive Primary Care Plus. *JAMA Netw Open*. Published April 26, 2024.
doi:10.1001/jamanetworkopen.2024.8519

Data
Data available: No

Additional Information
Explanation for why data not available: The data is only accessible via a data use agreement with Independence Blue Cross.
